# Association between circadian rhythm sleep disorder and open-angle glaucoma: The modifying role of melatonin

**DOI:** 10.21203/rs.3.rs-9740601/v1

**Published:** 2026-06-28

**Authors:** TAHER ELEIWA, MUHAMMAD CHAUHAN, KRISHNA KISHOR, SUBHI AL AREF, Mohamed Khodeiry, IBRAHIM ABBOUD, Sanjoy Bhattacharya, RICHARD LEE, ABDELRAHMAN ELHUSSEINY

**Affiliations:** Benha University; University of Arkansas for Medical Sciences; University of Miami; University of Arkansas for Medical Sciences; University of Louisville; University of California, Riverside; University of Miami; University of Miami; University of Arkansas for Medical Sciences

**Keywords:** Glaucoma, open angle, sleep, circadian rhythm, melatonin

## Abstract

**Background:**

Circadian rhythm sleep disorders (CRSD) may disrupt neurovascular regulation and increase open-angle glaucoma (OAG) and ocular hypertension (OHT) risk. This study assessed associations with OAG, primary open-angle glaucoma (POAG), and OHT and examined melatonin’s modifying effect.

**Methods:**

A retrospective cohort study using TriNetX data included adults (≥ 18 years) with CRSD matched 1:1 to polysomnography-tested controls without sleep disorders. Melatonin subgroups were analyzed. Among 24,730 matched pairs, ICD-10–defined OAG, POAG, and OHT at 1, 3, and 5 years were assessed using adjusted hazard ratios (aHRs) and Kaplan–Meier analyses.

**Results:**

After matching, 24,730 patients were included in each group. The CRSD group had a significantly higher risk of OAG at 1 year (0.14% vs. 0.04%; P=.0005; aHR 2.67, 95% CI 1.42–5.02), 3 years (0.25% vs. 0.07%; P < .0001; aHR 3.02, 95% CI 1.81–5.05), and 5 years (0.27% vs. 0.08%; P < .0001; aHR 2.88, 95% CI 1.78–4.66). Similar trends were observed for POAG, while no significant differences were seen for OHT. Among CRSD patients not using melatonin (n = 24,150 pairs), OAG risk remained significantly elevated at all time points. In contrast, among melatonin users (n = 4,081 pairs), the risks of OAG, and OHT were not significantly different from controls (5-year OAG: 0.52% vs. 0.32%; P = .23; aHR 2.07, 95% CI 0.88–4.84).

**Conclusions:**

CRSD is associated with an increased risk of OAG and POAG, in patients not using melatonin. Melatonin use appears to decrease this risk, suggesting a potential protective effect.

## Introduction

Circadian Rhythm Sleep Disorders (CRSD) are characterized by disruptions in the sleep-wake cycle, leading to misalignment between an individual’s internal circadian clock and the external environment.^1^ CRSD includes several subtypes, such as delayed sleep-wake phase disorder, advanced sleep-wake phase disorder, irregular sleep-wake rhythm disorder, and non-24-hour sleep-wake disorder, each defined by distinct patterns of sleep timing abnormalities.^2^ Emerging evidence suggests that circadian dysregulation may influence ocular physiology, including intraocular pressure (IOP) fluctuations, retinal ganglion cell (RGC) health, and vascular homeostasis; factors implicated in the pathogenesis of open-angle glaucoma (OAG) and ocular hypertension (OHT).^3,4^

Glaucoma, a leading etiology of irreversible blindness worldwide, affects over 76 million people, with projections suggesting an increase to over 111 million by 2040, posing a significant public health burden due to its asymptomatic progression and vision-threatening consequences.^5^ Notably, intrinsically photosensitive retinal ganglion cells (ipRGCs), which play a critical role in circadian regulation, are vulnerable in glaucomatous optic neuropathy, suggesting a potential bidirectional relationship between sleep disorders and glaucoma.^6^

Melatonin, a key circadian regulator, has demonstrated both neuroprotective and IOP-lowering effects in experimental models, yet its role in modulating glaucoma risk among individuals with CRSD remains unclear.^3,7^ Understanding this association could have important clinical implications for glaucoma prevention and management in populations with circadian disruptions.

This study aimed to investigate the association between CRSD and the risk of developing OAG, primary open-angle glaucoma (POAG), and OHT, with an additional focus on the potential effect of melatonin use.

## Materials and Methods

This retrospective cohort study utilized anonymized patient data from the TriNetX United States Collaborative Network, a comprehensive real-world data resource encompassing over 100 million deidentified patients. Data were sourced from 68 US healthcare organizations (HCOs), including demographic, clinical, and laboratory information, and were accessed in December 2024. Ethics approval and informed consent were waived by the Institutional Review Board of the University of Arkansas for Medical Sciences (UAMS) because this study used fully de-identified data obtained from the TriNetX research network. The dataset was fully anonymized and compliant with the Health Insurance Portability and Accountability Act (HIPAA), and all procedures adhered to the ethical principles of the Declaration of Helsinki.

Patients aged ≥ 18 years or older with a documented diagnosis of CRSD were included in the study. We had 4 study groups: (1) CRSD (overall); (2) CRSD plus melatonin; (3) CRSD minus melatonin; (4) control (no CRSD). For the first group, we included the whole cohort of patients with a diagnosis of CRSD using the ICD-10 code G47.2. We excluded patients with a prior diagnosis of insomnia, OAG, OHT, glaucoma suspect, or any glaucoma status at or before the CRSD-coded diagnosis. The second and third groups (subgroups of the first group) included patients with CRSD, along with use or without use of melatonin (RxNorm: 6711). The control group consisted of individuals who underwent polysomnography (CPT: 95808, 95811, and 95810) without a diagnosis of CRSD (ICD-10: G47.2), sleep deprivation (ICD-10: Z72.820), or morbid obesity (ICD-10: E66.2), along with additional exclusion criteria as the study groups.

The index date was defined as the first documented diagnosis of CRSD, while for controls, it was assigned to correspond with the day of polysomnography. All patients were required to have at least one eye exam during the study period to be included in the analysis.

To reduce potential confounding, 1:1 propensity score matching (PSM) was performed using a nearestneighbor matching algorithm. The propensity score was calculated based on a comprehensive set of covariates, including demographic characteristics, comorbid conditions, laboratory findings, and systemic medication use. Demographic variables included age at the index date, sex, race (White, Black or African American, Asian), and ethnicity (Hispanic, Non-Hispanic). Comorbidities considered for matching included hypertensive disease, hyperlipidemia, diabetes mellitus, thyroid disorders, chronic lower respiratory diseases, migraine, ischemic heart diseases, cerebrovascular diseases, and chronic kidney disease.

The effectiveness of the PSM was assessed by comparing standardized mean differences (SMDs) for covariates before and after matching, with an SMD of less than 0.1 considered indicative of adequate balance. Following PSM, baseline characteristics were summarized using descriptive statistics, with continuous variables presented as means with standard deviations and categorical variables as counts and percentages.

The primary clinical endpoints were the development of OHT (ICD-10: H40.05X) and OAG (ICD-10: H40.11X) at one, three, and five years following the index date. Adjusted hazard ratios (aHRs) with 95% confidence intervals (CIs) were calculated. The proportional hazards assumption was assessed using the generalized Schoenfeld method. Kaplan-Meier survival analyses were performed to estimate time-to-event probabilities for OHT and OAG within the matched cohorts, and the log-rank test was used to compare survival distributions between groups. All statistical analyses were conducted using the TriNetX analytics platform, and statistical significance was defined as a p-value of less than 0.05.

## Results

### Circadian Rhythm Sleep Disorder Group versus the Control Group

Before PSM, the study cohorts consisted of (1) 54,740 patients who were diagnosed with CRSD, of whom 4,641 were on melatonin and 50,099 were not on melatonin (2) 25,575 patients in the control group. Among those with CRSD, the proportion of female patients was ~ 54% (versus ~ 52% in the control group). There were 69% Whites in the CRSD group versus 57% in the control group. The mean ages at the index event were 39.49 (± 19.76) years for the CRSD versus 41.71 (± 20.68) years for the control group. After applying PSM, equally sized groups were matched for analysis with 24,730 patients in each group (Table 1).

**Figure 1:**
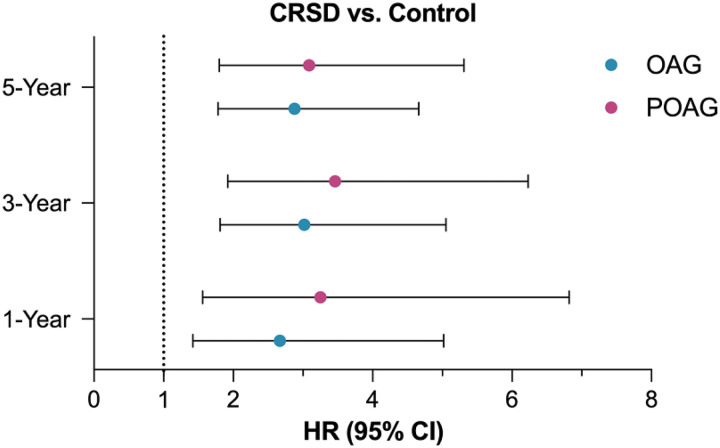
Adjusted hazard ratio (HR) for developing open-angle glaucoma (OAG) and primary open-angle glaucoma (POAG) in the circadian rhythm sleep disorder (CRSD) cohort compared with matched controls at 1, 3, and 5-year follow-up after propensity score matching.

The risk of OAG was significantly higher in the CRSD group at one year (0.14% vs.0.04% in the control group, p = 0.0005), three years (0.25% vs.0.07% in the control group, p < 0.0001), and five years (0.27% vs. 0.08% in the control group, p < 0.0001). Similarly, the risk of POAG was significantly higher in the CRSD group at one year (0.11% vs. 0.03% in the control group, p = 0.0007), three years (0.21% vs.0.05% in the control group, p < 0.0001), and five years (0.22% vs. 0.06% in the control group, p < 0.0001). For OHT, none of the patients in either group developed OHT at one year. At three and five years, there were no significant differences between the groups (0.039% in each group at three and five years). We found an increased risk of OAG at one year with aHR of 2.67 (95% CI: 1.42–5.02). The aHR at three years was 3.02 (95% CI: 1.81–5.05), and at five years was 2.88 (95% CI: 1.78–4.66). For the POAG subgroup, the aHRs were 3.25 (95% CI: 1.55–6.82) at one year, 3.46 (95% CI: 1.92–6.23) at three years, and 3.09 (95% CI: 1.80–5.31) at five years (Fig. 1).

### Circadian Rhythm Sleep Disorder Patients Not Using Melatonin versus the Control Group

After applying PSM, 24,150 patients with CRSD not using melatonin were matched with an equally sized group of control patients (Table 2). Among the no melatonin CRSD group patients, the age at the index event was 39.00 (± 19.14) years, and the majority were females (54.55%) and Whites (69.66%).

**Figure 2:**
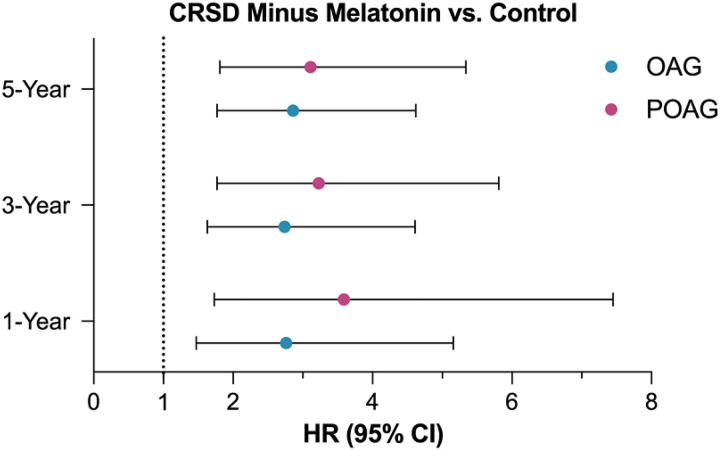
Adjusted hazard ratio (HR) for developing open-angle glaucoma (OAG) and primary open-angle glaucoma (POAG) in the circadian rhythm sleep disorder (CRSD) cohort not using melatonin compared with matched controls at 1, 3, and 5-year follow-up after propensity score matching.

The risk of OAG was significantly higher in the no melatonin CRSD group at one year (0.16% vs.0.05% in the control group, p = 0.0002), three years (0.23% vs.0.07% in the control group, p < 0.0001), and five years (0.27% vs. 0.08% in the control group, p < 0.0001). Similarly, the risk of POAG was significantly higher in the CRSD group at one year (0.14% vs. 0.04% in the control group, p = 0.0001), three years (0.19% vs.0.05% in the control group, p < 0.0001), and five years (0.23% vs. 0.06% in the control group, p < 0.0001). For OHT, none of the patients in either group developed OHT at one year. At three and five years, there were no significant differences between the groups [0.04% in each group at three (p = 0.99) and five years (p = 0.99)]. We found an increased risk of OAG at one year with aHR of 2.76 (95% CI: 1.47–5.16). The aHR at three years was 2.74 (95% CI: 1.63–4.61) and at five years was 2.86 (95% CI: 1.77–4.62). For the POAG subgroup, the aHRs were 3.59 (95% CI: 1.73–7.45) at one year, 3.23 (95% CI: 1.77–5.81) at three years, and 3.11 (95% CI: 1.81–5.34) at five years (Fig. 2).

### Circadian Rhythm Sleep Disorder Patients Using Melatonin versus the Control Group

After applying PSM, 4,081 patients with CRSD using melatonin were matched with an equally sized group of control patients (Table 3). Among the melatonin group patients, the age at the index event was 46.76 (± 24.52) years, and the majority were females (51.07%) and Whites (67.38%).

**Figure 3:**
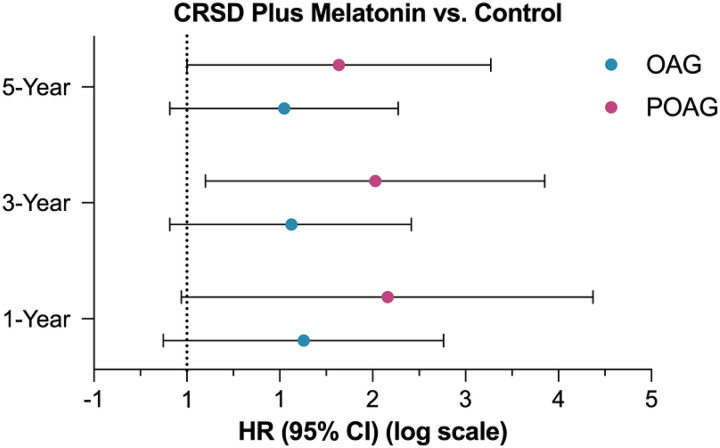
Adjusted hazard ratio (HR) for developing open-angle glaucoma (OAG) and primary open-angle glaucoma (POAG) in the circadian rhythm sleep disorder (CRSD) cohort using melatonin compared with matched controls at 1, 3, and 5-year follow-up after propensity score matching.

There were no significant differences in the risks of OAG between the melatonin group and the control group at one year (0.39% vs.0.32% in the control group, p = 0.66), three years (0.49% vs.0.32% in the control group, p = 0.31), and five years (0.52% vs. 0.32% in the control group, p = 0.23). Similarly, the risks of POAG were not significantly different between both groups at one year (0.32% vs. 0.32% in the control group, p = 1.00), three years (0.39% vs.0.32% in the control group, p = 0.66), and five years (0.39% vs. 0.32% in the control group, p = 0.66). Additionally, there were no significant differences in the risks of OHT at any time point. We found no significant difference in the risk of OAG at one year (aHR: 2.39, 95% CI: 0.84–6.79), three years (aHR: 2.18, 95% CI: 0.88–5.34), or at five years (aHR: 2.07, 95% CI: 0.88–4.84). For the POAG subgroup, the aHRs were 4.47 (95% CI: 0.96–20.71) at one year, 4.08 (95% CI: 1.15–14.45) at three years, and 3.11 (95% CI: 1.00–9.66) at five years (Fig. 3).

## Discussion

We observed a significant association between CRSD and an increased risk of developing OAG and POAG by around 3-fold. Particularly, this association was pronounced among CRSD patients not using melatonin (a 3-fold increase in risk), while no significant differences in glaucoma risk were observed among CRSD patients who used melatonin. Our findings provide new insights into the potential interplay between circadian dysregulation, melatonin supplementation, and glaucoma pathogenesis.

IOP is well known to follow a diurnal rhythm, tending to peak during nocturnal or early morning hours in many individuals.^8^ Sleep and circadian patterns modulate this rhythm – for example, posture and systemic physiology during sleep can alter IOP.^9^ A person with a misaligned or irregular circadian cycle (as in with CRSD) may experience abnormal timing or magnitude of IOP fluctuations. These fluctuations, especially if they occur during vulnerable periods (for example, during a high IOP spike during the night when optic nerve perfusion is lowest), could facilitate glaucomatous damage even if daytime IOP appears normal.^10,11^ Prior research supports that large swings in IOP or a disrupted circadian IOP pattern can worsen optic nerve injury – one study noted that a disturbed 24-hour IOP rhythm (with higher nocturnal IOP) was associated with greater retinal ganglion cell loss in glaucoma.^12^ Moreover, the “vascular theory” of glaucoma provides a complementary explanation: chronic circadian rhythm disorders might impair the autonomic regulation of blood pressure and ocular blood flow. Nocturnal systemic hypotension (an exaggerated dip in blood pressure during sleep) and other vascular dysregulation phenomena have been linked to glaucoma development and progression, particularly in normal-tension glaucoma.^13^ Indeed, patients with systemic conditions like extreme nighttime blood pressure dips, obstructive sleep apnea (OSA), migraines, or Raynaud syndrome – many of which involve autonomic dysfunction – have higher glaucoma risk. In our CRSD cohort, chronic irregular sleep-wake cycles and misaligned light exposure could similarly provoke dysregulated sympathetic activity or loss of the normal nocturnal blood pressure profile, thereby reducing optic nerve perfusion. Supporting this concept, investigators have shown that reduced ocular perfusion pressure has a deleterious effect on the optic nerve, particularly at night when compensatory vascular mechanisms may fail.^14^ Furthermore, OSA – a distinct sleep disorder often considered separately from CRSD – is well documented to increase glaucoma risk, presumably through intermittent hypoxia and surges in blood pressure that injure retinal ganglion cells.^15^ While our study excluded OSA as a primary exposure, these examples illustrate how sleep/circadian disturbances can create an unfavorable milieu for the optic nerve through IOP and vascular pathways. Taken together, the mechanistic picture suggests that circadian disruption may synergistically affect both IOP fluctuations and ocular blood flow – the two principal drivers of glaucomatous optic neuropathy.

Our stratified analysis offers intriguing insight into melatonin’s potential impact. CRSD patients who were not on melatonin had a significantly elevated risk of OAG/POAG versus controls, whereas those patients with CRSD who were documented as using melatonin did not show a statistically significant increase in glaucoma incidence. These findings suggest the possibility that exogenous melatonin therapy may mitigate the deleterious ocular effects of circadian disruption. This would be biologically plausible as pharmacologic melatonin has been shown to decrease IOP in both normotensive and hypertensive eyes by acting on the melatonin receptors in the ciliary processes.^16^ In experimental models and clinical studies, melatonin administration can also reduce the amplitude of IOP fluctuations and improve retinal ganglion cell function. For example, a recent interventional study in glaucoma patients reported that nightly melatonin not only lowered IOP variability but also increased the electrophysiologic responsiveness of RGCs in individuals with advanced glaucoma.^17^ Melatonin is thought to exert dual benefits – acutely reducing IOP and also protecting RGCs via anti-oxidative and anti-apoptotic pathways in the context of neurodegeneration. Additionally, by reinforcing the alignment of circadian rhythms, melatonin might counteract some systemic effects of circadian disruption that could impact glaucoma. Previous work has found that exogenous melatonin helped resynchronize circadian rhythms.^18^ This internal synchronization may reduce stress on the optic nerve over the daily cycle.^18^ Our findings also align with evidence that circadian misalignment in glaucoma patients is associated with sleep disturbances and mood disorders, highlighting the systemic impact of glaucoma beyond vision loss. This underscores the need for a holistic approach to glaucoma management, addressing both ocular and systemic health. Considering circadian health in glaucoma patients may improve not only disease outcomes but also overall quality of life.

Several limitations merit emphasis. First, outcome ascertainment relied on diagnosis codes (H40.11X for POAG; H40.05X for OHT) rather than chart-adjudicated glaucoma with standardized structural/functional criteria; under-ascertainment likely contributed to the low absolute event rates. Second, we could not capture IOP measurements, nocturnal IOP profiles, central corneal thickness, family history, refractive errors, or optic nerve imaging—factors that would refine risk attribution. Third, although we matched widely on systemic comorbidities, residual confounding is possible (e.g., sleep apnea severity and treatment, nocturnal hypotension, topical/systemic steroid exposure, and race-specific glaucoma risk patterns). Fourth, “CRSD” aggregates heterogeneous subtypes (delayed sleep-wake, advanced sleep-wake, non-24-hour, irregular sleep-wake, shift-work), which may differentially influence ocular physiology; granular subtype analyses were not feasible. Fifth, melatonin exposure is incompletely captured in EHRs because it is often used over-the-counter; misclassification would bias any true association toward the null. Future prospective studies and randomized controlled trials are needed to confirm these findings and elucidate the mechanisms underlying the protective effects of melatonin in glaucoma. Investigating the role of circadian rhythm stabilization strategies, including light therapy and chronobiological interventions, may also provide novel insights into glaucoma prevention and management.

In a large, real-world cohort, clinically diagnosed CRSD was associated with an approximately three-fold higher hazard of developing OAG/POAG over 1–5 years, without a corresponding increase in coded OHT. The signal was driven by patients with CRSD not receiving documented melatonin, while findings among those with recorded melatonin use were inconclusive because of small numbers and likely exposure under-capture. These results strengthen the case that circadian dysregulation is relevant to glaucoma pathogenesis and support incorporating circadian considerations into glaucoma risk assessment and future interventional research.

## Figures and Tables

**Table 1:** Baseline characteristics of the circadian rhythm sleep disorder (CRSD) cohort and matched control cohort before and after propensity score matching.

Characteristic	Before Matching			After Matching		
	CRSD (%)	Control (%)	Standard Difference	CRSD (%)	Control (%)	Standard Difference
	n = 54740	n = 25575		n = 24730	n = 24730	
**Demographics**						
**Age at Index**	39.49 (± 19.76)	41.71 (± 20.68)	0.110	42.35 (± 20.34)	41.54 (± 20.67)	0.039
**Male**	45.70%	47.17%	0.029	47.83%	47.32%	0.010
**Female**	54.26%	52.56%	0.034	52.08%	52.54%	0.009
**Hispanic or Latino**	*7.22%*	14.44%	0.234	13.04%	13.18%	0.004
**Not Hispanic or Latino**	71.94%	62.76%	0.197	62.98%	63.65%	0.014
**White**	69.43%	57.31%	0.254	58.66%	58.80%	0.003
**Black or African American**	12.38%	19.34%	0.191	18.56%	18.65%	0.002
**Asian**	3.52%	1.49%	0.130	1.61%	1.54%	0.006
**American Indian or Alaska Native**	0.42%	0.51%	0.014	0.52%	0.51%	0.001
**Native Hawaiian or Other Pacific Islander**	0.32%	0.38%	0.009	0.46%	0.38%	0.013
**Other Race**	4.75%	8.41%	0.148	7.51%	7.70%	0.007
**Diagnoses**						
**Hypertensive diseases (I10-I1A)**	18.66%	19.73%	0.027	19.97%	19.33%	0.016
**Ischemic heart diseases (I20-I25)**	4.58%	4.88%	0.014	4.87%	4.77%	0.005
**Cerebrovascular diseases (I60-I69)**	4.49%	3.24%	0.065	3.22%	3.29%	0.004
**Chronic kidney disease (CKD) (N18)**	2.91%	2.44%	0.029	2.47%	2.41%	0.003
**Diabetes mellitus (E08-E13)**	6.87%	7.50%	0.024	7.36%	7.28%	0.003
**Overweight and obesity (E66)**	12.59%	17.23%	0.131	16.92%	16.39%	0.014
**Disorders of lipoprotein metabolism and other lipidemias (E78)**	18.12%	15.82%	0.061	16.28%	15.85%	0.012
**Hyperlipidemia, unspecified (E78.5)**	12.29%	10.66%	0.051	11.08%	10.67%	0.013
**Sleep apnea (G47.3)**	2.93%	0.34%	0.205	0.49%	0.35%	0.022
**Chronic lower respiratory diseases (J40-J4A)**	14.37%	13.61%	0.022	13.66%	13.41%	0.008
**Disorders of thyroid gland (E00-E07)**	9.42%	7.15%	0.082	7.41%	7.23%	0.007
**Nicotine dependence (F17)**	7.27%	6.08%	0.048	6.23%	6.09%	0.006
**Migraine (G43)**	8.69%	5.40%	0.129	5.69%	5.52%	0.008
**Myopia (H52.1)**	3.33%	1.48%	0.121	1.65%	1.53%	0.009
**Degenerative myopia (H44.2)**	0.07%	0.04%	0.015	0.04%	0.04%	0.000
**Persons with health hazards related to socioeconomic and psychosocial circumstances (Z55-Z65)**	3.19%	1.24%	0.132	1.42%	1.28%	0.012
**Procedures**						
**Assistance with Respiratory Ventilation (5A09357)**	0.20%	0.49%	0.049	0.36%	0.39%	0.004
**Extracapsular cataract removal - routine (66984)**	0.34%	0.28%	0.011	0.30%	0.28%	0.002
**Extracapsular cataract removal – complex (66982)**	0.06%	0.04%	0.008	0.06%	0.04%	0.009
**Ophthalmology Services and Procedures (1012793)**	6.16%	4.24%	0.087	4.25%	4.31%	0.003
**Medications**						
**Corticosteroids (R01AD)**	30.19%	29.94%	0.006	29.92%	29.53%	0.008
**Beta Blockers (C07)**	13.20%	15.42%	0.063	14.56%	14.82%	0.007
**HMG CoA reductase inhibitors (C10AA)**	11.58%	14.57%	0.089	13.79%	13.91%	0.003
**Calcium Channel blockers (C08)**	7.98%	11.36%	0.115	10.66%	10.57%	0.003

**Table 2:** Baseline characteristics of the circadian rhythm sleep disorder (CRSD) cohort not using melatonin and matched control cohort before and after propensity score matching.

Characteristic	Before Matching			After Matching		
	CRSD Minus Melatonin (%) n = 50099	Control (%) n = 25575	Standard Difference	CRSD Minus Melatonin (%) n = 24150	Control (%) n = 24150	Standard Difference
**Demographics**						
**Age at Index**	39.00 (± 19.14)	41.71 (± 20.68)	0.136	41.95 (± 19.97)	41.35 (± 20.65)	0.030
**Male**	45.41%	47.17%	0.035	47.98%	47.29%	0.014
**Female**	54.55%	52.56%	0.040	51.94%	52.58%	0.013
**Hispanic or Latino**	7.13%	14.44%	0.237	12.54%	12.64%	0.003
**Not Hispanic or Latino**	71.47%	62.76%	0.186	62.61%	64.03%	0.029
**White**	69.66%	57.31%	0.259	59.19%	59.73%	0.011
**Black or African American**	11.87%	19.34%	0.207	18.14%	17.98%	0.004
**Asian**	3.53%	1.49%	0.130	1.59%	1.58%	0.001
**American Indian or Alaska Native**	0.42%	0.51%	0.013	0.45%	0.51%	0.009
**Native Hawaiian or Other Pacific Islander**	0.30%	0.38%	0.013	0.39%	0.38%	0.001
**Other Race**	4.75%	8.41%	0.148	7.35%	7.42%	0.003
**Diagnoses**						
**Hypertensive diseases (I10-I1A)**	17.31%	19.73%	0.062	19.33%	18.98%	0.009
**Ischemic heart diseases (I20-I25)**	3.83%	4.88%	0.051	4.60%	4.59%	0.001
**Cerebrovascular diseases (I60-I69)**	3.43%	3.24%	0.011	3.26%	3.18%	0.004
**Chronic kidney disease (CKD) (N18)**	2.35%	2.44%	0.006	2.46%	2.36%	0.007
**Demographics**						
**Diabetes mellitus (E08-E13)**	6.22%	7.50%	0.050	7.25%	7.13%	0.005
**Overweight and obesity (E66)**	12.22%	17.23%	0.142	16.44%	15.98%	0.012
**Disorders of lipoprotein metabolism and other lipidemias (E78)**	17.13%	15.82%	0.035	16.14%	15.75%	0.011
**Hyperlipidemia, unspecified (E78.5)**	11.35%	10.66%	0.022	10.98%	10.61%	0.012
**Sleep apnea (G47.3)**	2.93%	0.34%	0.205	0.49%	0.36%	0.020
**Chronic lower respiratory diseases (J40-J4A)**	13.72%	13.61%	0.003	13.32%	13.33%	0.000
**Disorders of thyroid gland (E00-E07)**	8.98%	7.15%	0.067	7.30%	7.29%	0.000
**Nicotine dependence(F17)**	6.66%	6.08%	0.024	6.18%	6.09%	0.004
**Migraine (G43)**	8.69%	5.40%	0.128	5.81%	5.60%	0.009
**Myopia (H52.1)**	3.26%	1.48%	0.117	1.61%	1.56%	0.004
**Degenerative myopia (H44.2)**	0.07%	0.04%	0.013	0.04%	0.04%	0.000
**Persons with health hazards related to socioeconomic and psychosocial circumstances (Z55-Z65)**	2.87%	1.24%	0.115	1.55%	1.31%	0.020
**Procedures**						
**Assistance with Respiratory Ventilation (5A09357)**	0.29%	0.28%	0.002	0.29%	0.27%	0.004
**Extracapsular cataract removal - routine (66984)**	0.15%	0.49%	0.061	0.27%	0.33%	0.011
**Extracapsular cataract removal - complex (66982)**	0.05%	0.04%	0.003	0.05%	0.04%	0.002
**Ophthalmology Services and Procedures (1012793)**	5.97%	4.24%	0.079	4.49%	4.32%	0.008
**Medications**						
**Corticosteroids (R01AD)**	28.98%	29.94%	0.021	29.76%	29.14%	0.014
**Beta Blockers (C07)**	11.74%	15.42%	0.108	14.12%	14.26%	0.004
**HMG CoA reductase inhibitors (C10AA)**	10.40%	14.57%	0.127	13.39%	13.44%	0.002
**Calcium Channel blockers (C08)**	7.06%	11.36%	0.149	10.01%	10.07%	0.002

**Table 3:** Baseline characteristics of the circadian rhythm sleep disorder (CRSD) cohort using melatonin and matched control cohort before and after propensity score matching.

Characteristic	Before Matching			After Matching		
	CRSD Melatonin (%) n = 4641	Control (%) n = 25575	Standard Difference	CRSD Melatonin (%) n = 4081	Control (%) n = 4081	Standard Difference
**Demographics**						
**Age at Index**	46.76 (± 24.52)	41.71 (± 20.68)	0.223	45.08 (± 24.27)	43.14 (± 21.38)	0.085
**Male**	48.89%	47.17%	0.034	48.42%	47.93%	0.010
**Female**	51.07%	52.56%	0.030	51.53%	52.02%	0.010
**Hispanic or Latino**	8.06%	14.44%	0.203	8.72%	9.16%	0.015
**Not Hispanic or Latino**	76.38%	62.76%	0.299	75.15%	75.40%	0.006
**White**	67.38%	57.31%	0.209	66.55%	66.28%	0.006
**Black or African American**	17.24%	19.34%	0.054	17.59%	17.20%	0.010
**Asian**	3.23%	1.49%	0.115	2.92%	3.21%	0.017
**American Indian or Alaska Native**	0.39%	0.38%	0.002	0.37%	0.37%	0.000
**Native Hawaiian or Other Pacific Islander**	0.32%	0.51%	0.029	0.34%	0.25%	0.018
**Other Race**	4.89%	8.41%	0.142	5.22%	5.51%	0.013
**Diagnoses**						
**Hypertensive diseases (I10-I1A)**	40.53%	19.73%	0.465	36.14%	35.92%	0.005
**Ischemic heart diseases (I20-I25)**	15.88%	4.88%	0.367	12.62%	12.42%	0.006
**Cerebrovascular diseases (I60-I69)**	10.99%	5.40%	0.205	10.24%	10.51%	0.009
**Chronic kidney disease (CKD) (N18)**	11.61%	2.44%	0.365	8.48%	8.28%	0.007
**Diabetes mellitus (E08-E13)**	17.82%	7.15%	0.327	15.24%	15.17%	0.002
**Overweight and obesity (E66)**	35.83%	15.82%	0.470	31.73%	32.37%	0.014
**Disorders of lipoprotein metabolism and other lipidemias (E78)**	28.40%	10.66%	0.459	24.50%	25.17%	0.015
**Hyperlipidemia, unspecified (E78.5)**	21.66%	17.23%	0.112	20.58%	20.95%	0.009
**Sleep apnea (G47.3)**	5.37%	0.34%	0.305	2.06%	1.79%	0.020
**Chronic lower respiratory diseases (J40-J4A)**	25.06%	13.61%	0.293	23.06%	22.27%	0.019
**Disorders of thyroid gland (E00-E07)**	17.56%	7.50%	0.308	15.27%	15.09%	0.005
**Nicotine dependence(F17)**	17.54%	6.08%	0.361	15.19%	15.93%	0.020
**Migraine (G43)**	19.24%	3.24%	0.524	14.34%	14.21%	0.004
**Myopia (H52.1)**	8.94%	1.24%	0.356	5.91%	6.25%	0.014
**Degenerative myopia (H44.2)**	0.22%	0.04%	0.049	0.25%	0.25%	0.000
**Persons with health hazards related to socioeconomic and psychosocial circumstances (Z55-Z65)**	4.68%	1.48%	0.186	4.12%	4.48%	0.018
**Procedures**						
**Assistance with Respiratory Ventilation (5A09357)**	1.10%	0.49%	0.069	1.03%	1.18%	0.014
**Extracapsular cataract removal - routine (66984)**	1.29%	0.28%	0.115	0.93%	0.98%	0.005
**Extracapsular cataract removal - complex (66982)**	0.28%	0.04%	0.059	0.25%	0.25%	0.000
**Ophthalmology Services and Procedures (1012793)**	9.85%	4.24%	0.221	8.85%	9.68%	0.029
**Medications**						
**Corticosteroids (R01AD)**	50.77%	29.94%	0.434	47.54%	46.36%	0.024
**Beta Blockers (C07)**	35.14%	15.42%	0.466	31.02%	30.70%	0.007
**HMG CoA reductase inhibitors (C10AA)**	28.81%	14.57%	0.351	25.48%	25.17%	0.007
**Calcium Channel blockers (C08)**	22.24%	11.36%	0.294	19.60%	19.02%	0.015

## Data Availability

The datasets generated and/or analyzed during the current study are available in the TriNetX research network repository, https://trinetx.com. The data are not publicly available as they are from a licensed subscription-based platform containing proprietary electronic health records. Access is restricted to authorized users at participating institutions, and therefore, no accession numbers are available.

## Summary Box

### What was known before

- Circadian rhythm sleep disorders (CRSD) are associated with systemic neurovascular dysregulation and abnormal intraocular pressure rhythms, mechanisms implicated in glaucoma pathogenesis.
- Prior studies suggested links between sleep disorders and glaucoma, but population-level data specifically evaluating CRSD and longitudinal glaucoma risk were limited.
- Melatonin has demonstrated intraocular pressure–lowering and neuroprotective effects in experimental and small clinical studies, but its modifying role in glaucoma risk among CRSD patients was unclear.

### What this study adds

- In a large, propensity-matched cohort, CRSD was associated with an approximately three-fold increased risk of developing open-angle glaucoma and primary open-angle glaucoma over 1–5 years.
- This increased risk was driven by CRSD patients not using melatonin, while CRSD patients with documented melatonin use did not show a statistically significant increase in glaucoma risk.
- The findings suggest circadian dysregulation as a clinically relevant glaucoma risk factor and support further investigation of melatonin and circadian stabilization strategies in glaucoma prevention.
